# Antibody-based proteomics to identify an apoptosis signature for early recurrence of hepatocellular carcinoma

**DOI:** 10.1186/s12014-016-9130-0

**Published:** 2016-10-24

**Authors:** Noriaki Morofuji, Hidenori Ojima, Nobuyoshi Hiraoka, Takuji Okusaka, Minoru Esaki, Satoshi Nara, Kazuaki Shimada, Yoshiro Kishi, Tadashi Kondo

**Affiliations:** 1 Division of Rare Cancer Research, National Cancer Center Research Institute, 5-1-1 Tsukiji, Chuo-ku, Tokyo, 104-0045 Japan; 2 Pathology Division, National Cancer Center Research Institute, Tokyo, 104-0045 Japan; 3 Hepatobiliary and Pancreatic Oncology Division, National Cancer Center Hospital, Tokyo, 104-0045 Japan; 4 Hepatobiliary and Pancreatic Surgery Division, National Cancer Center Hospital, Tokyo, 104-0045 Japan; 5 Research and Development Division, Molecular and Biological Laboratories, Nagano, 396-0002 Japan; 6 Department of Surgery, Kugayama Hospital, 2-14-20 Kitakarasuyama, Setagaya-ku, Tokyo, 154-0061 Japan; 7 Department of Pathology, School of Medicine, Keio University, 35 Shinanomachi, Shinjuku-ku, Tokyo, 160-0016 Japan

**Keywords:** Antibody-based proteomics, Apoptosis, Biomarker, Early recurrence, Hepatocellular carcinoma

## Abstract

**Background:**

Early recurrence after surgical resection is a hallmark of poor prognosis in hepatocellular carcinoma (HCC). To determine the proteomic background of early recurrence of HCC, we focused on apoptosis-related proteins.

**Methods:**

Surgically resected tumor tissues were obtained from 80 patients, including HCC tumor tissues, non-tumor tissues, and normal liver tissues. These samples were grouped in the discovery and validation sample sets. The expression level of 192 apoptosis-related proteins was monitored using 247 commercially available antibodies and western blotting. The intensity of protein bands was compared between the tumor and non-tumor tissues as well as between the patients who had recurrence within 2 years after surgery and those who did not.

**Results:**

In the first screening, we used pooled samples. The intensity of 53 protein bands detected by 37 unique antibodies was higher in tumor tissues compared with normal liver tissues, especially tumor tissues from patients who had recurrence within 2 years after surgery. In the second screening, we examined individual samples used to make the pooled samples. Among the selected bands and antibodies, the intensity of 18 protein bands detected by 11 antibodies was higher in tumor tissues compared with that in normal tissues, especially tumor tissues from the patients with early recurrence after surgery. For the third screening, we examined the samples from newly enrolled patients using these 11 antibodies. Eighteen protein bands detected by six antibodies were selected by using the same criteria. The corresponding antigens included ERK1, PKG, Apaf1, BclX, phosphorylated c-abl, and PIASx1/2.

**Conclusions:**

We screened 192 apoptosis-related proteins using specific antibodies and western blotting. We identified 6 apoptosis-related proteins associated with carcinogenesis and early recurrence in HCC. The biological and clinical significance of the identified proteins are worth further investigation.

**Electronic supplementary material:**

The online version of this article (doi:10.1186/s12014-016-9130-0) contains supplementary material, which is available to authorized users.

## Background

Hepatocellular carcinoma (HCC) is a major public health problem with especially high prevalence in Asia and Africa [1]. Recent studies indicate that HCC incidence has increased substantially in the US and UK over the last decades [2–4]. Surgery is the only curative treatment, and fewer than 15% of patients undergo surgery because of late clinical presentation and diagnosis. Survival for surgically incurable patients with HCC has not increased significantly over the past 30 years and, even after surgical resection, the early recurrence prevents favorable clinical outcome after curative resection. As a consequence, the overall 5-year survival rate remains at <5% in HCC [5]. Other treatment options such as molecular-targeted therapies for patients with advanced or metastatic HCC are limited [6]. Therefore, novel diagnostic and therapeutic approaches or improvement of the existing ones have long been desired to improve the clinical outcome of patients with HCC.

Apoptosis is the major regulatory mechanism disturbed during carcinogenesis and cancer progression in HCC. Tumor cells are exposed to various apoptotic stimuli during the carcinogenesis and metastasis steps, and fully transformed metastatic tumor cells acquire resistance against apoptotic stress [7]. Aberrant regulation of apoptosis-related proteins has been observed in more aggressive HCC cells. For example, anti-apoptotic regulators such as B cell lymphoma (Bcl)-2 family proteins have been identified as therapeutic targets in HCC [8, 9]. The expression of X-linked inhibitor-of-apoptosis protein promoted resistance to apoptosis, enhanced the invasiveness of HCC cells, and was a biomarker for relapse in patients with HCC [10]. Apoptosis-associated receptors and ligands also play key roles in HCC progression and are considered as therapeutic targets [11]. These observations strongly suggest that further investigation of the apoptosis pathway may reveal novel candidate biomarkers and therapeutic targets. However, although the apoptosis pathway includes hundreds of proteins, only a limited number of proteins have been investigated in the context of HCC, and the clinical applicability of these proteins remained to be challenged.

Proteomic studies identified specific proteins with expression levels that are correlated with malignant features of HCC [12–18]. Those that are implicated in early recurrence may be useful in the clinic as prognostic biomarkers and drug targets. Separation-based proteomics technologies such as mass spectrometry and two-dimensional differential gel electrophoresis (2D-DIGE) generate protein expression profiles based on the nature of the proteins. These methods provide opportunities for identifying unexpected proteins as biomarkers and therapeutic targets. However, they do not fully cover certain molecular pathways. For example, we extensively examined protein expression in the surgically resected tissues from patients with HCC using mass spectrometry [18] and 2D-DIGE [15]. However, we found that the proteome data obtained in our previous studies did not cover most apoptosis-related proteins. The antibody-based proteomics is a powerful knowledge-based tool that allows investigators to examine the expression profiles of particular proteins and pathways.

In this study, we investigated the status of the apoptosis pathway in HCC using a panel of 247 commercial antibodies against 192 proteins in surgically resected liver tissues. The expression of 192 apoptosis-associated proteins was compared between non-tumor and tumor tissues as well as between tumor tissues from patients with or without early recurrence after surgery.

## Methods

### Clinical samples

Surgically resected HCC tissues and adjacent non-tumor tissues were obtained from patients at the National Cancer Center Hospital who underwent initial hepatic resection between December 1999 and May 2005. The study was reviewed and approved by the Institutional Review Board of the National Cancer Center. Written informed consent was obtained from all participants in this study. Normal liver tissues were obtained from patients with colorectal cancer who underwent hepatic resection for metastatic liver tumors during the same period. Patients with HCC received curative surgery, but did not receive preoperative therapy. Tumors were classified according to the International Union against Cancer Tumor-node-metastasis Criteria. For the expression study, samples were divided into four groups: primary tumor tissue from patients with HCC who showed recurrence within 2 years after surgery (Group S), tumor tissue from patients with HCC who did not show a relapse within 2 years after surgery (Group L), adjacent liver tissue from patients with HCC (Group A), and normal liver tissue from patients with colorectal cancer and liver metastasis (Group N). Samples were randomly divided into two groups for discovery and validation purposes. Detailed clinical and pathological data are summarized in Tables 1 and 2.

**Table 1:** Clinicopathological features of 40 cases for discovery purpose

Sample no.	Group	Age	Gender	Virus infection status	Child-Pugh classification	Adjacent liver tissue	AFP (ng/ml)	TNM stage^a^	Tumor number	Tumor size (cm)	Differentiation^b^	Portal vein invesion	Intrahepatic metastasis	Duration of recurrence (year)
1	S	63	M	None	A	CH	137.5	3B	Single	7.30	Por	Presence	Presence	0.53
2	S	27	M	HBV	A	CH	91,850	2	Multiple	10.00	Por	Presence	Absence	0.63
3	S	72	M	None	A	CH	8061	2	Single	8.50	Por	Presence	Absence	0.89
4	S	29	F	HBV	A	Normal	56,570	2	Single	14.50	Por	Presence	Absence	1.84
5	S	70	M	HBV	A	CH	965.9	2	Multiple	3.30	Por	Presence	Absence	0.52
6	S	52	F	HBV	B	CH	57,820	3B	Single	11.00	Por	Presence	Presence	0.18
7	S	44	M	HBV	A	CH	2664	2	Single	4.50	Por	Presence	Presence	0.71
8	S	72	M	None	A	CH	107,890	2	Single	10.00	Por	Presence	Presence	0.27
9	S	68	M	HBV	A	CH	4739	2	Single	12.50	Por	Presence	Presence	0.19
10	S	52	M	HBV	A	CH	526	2	Single	7.30	Mod	Presence	Presence	0.50
11	L	71	M	None	A	CH	4.5	1	Single	3.50	Mod	Absence	Absence	3.33
12	L	51	M	HBV	A	CH	18.5	1	Single	12.00	Mod	Absence	Absence	3.07
13	L	62	M	None	A	CH	18.8	1	Single	2.80	Mod	Absence	Absence	4.96
14	L	73	M	HBV	A	CH	4.9	1	Single	4.50	Mod	Absence	Absence	2.99
15	L	64	M	HCV	A	CH	3.1	1	Multiple	2.10	Mod	Absence	Absence	7.08
16	L	72	M	HCV	A	LC	102.3	1	Multiple	3.00	Well	Absence	Absence	5.74
17	L	52	F	HBV	A	CH	12.2	1	Single	2.00	Mod	Absence	Absence	2.38
18	L	62	M	HCV	B	CH	5.5	1	Single	2.50	Mod	Absence	Absence	3.38
19	L	71	M	None	A	CH	3.2	1	Single	2.00	Mod	Absence	Absence	4.89
20	L	65	M	HCV	B	LC	34.1	1	Single	3.40	Mod	Absence	Absence	2.85
21	A	70	M	HCV	–	CH	–	–	–	–	–	–	–	–
22	A	79	M	HCV	–	CH	–	–	–	–	–	–	–	–
23	A	36	M	HBV	–	CH	–	–	–	–	–	–	–	–
24	A	57	M	HBV	–	LC	–	–	–	–	–	–	–	–
25	A	51	M	HBV	–	CH	–	–	–	–	–	–	–	–
26	A	53	M	None	–	LC	–	–	–	–	–	–	–	–
27	A	63	F	HCV	–	CH	–	–	–	–	–	–	–	–
28	A	61	M	HBV	–	LC	–	–	–	–	–	–	–	–
29	A	72	M	HCV	–	LC	–	–	–	–	–	–	–	–
30	A	52	F	HBV	–	CH	–	–	–	–	–	–	–	–
31	N	58	F	None	–	Normal	–	–	–	–	–	–	–	–
32	N	49	M	None	–	Normal	–	–	–	–	–	–	–	–
33	N	74	M	None	–	Normal	–	–	–	–	–	–	–	–
34	N	39	M	None	–	Normal	–	–	–	–	–	–	–	–
35	N	68	M	None	–	Normal	–	–	–	–	–	–	–	–
36	N	64	M	None	–	Normal	–	–	–	–	–	–	–	–
37	N	32	M	None	–	Normal	–	–	–	–	–	–	–	–
38	N	70	F	None	–	Normal	–	–	–	–	–	–	–	–
39	N	61	M	None	–	Normal	–	–	–	–	–	–	–	–
40	N	59	M	None	–	Normal	–	–	–	–	–	–	–	–

*S* duration of recurrence less than 2 years, *L* duration of recurrence more than 2 years, *A* adjacent liver tissue, *N* normal liver tissue, *HBV* hepatitis B virus, *HCV* hepatitis C virus, *CH* chronic hepatitis, *LC* liver cirrhosis

^a^TNM Classification of Malignant Tumours, 7th Edition, Sobin LH, Wittekind Ch (eds): International Union Against Cancer (UICC): ‘‘TNM classification of malignant tumors.’’ 7th ed. New York

^b^Well, well differentiated; Mod, moderately differentiated; Por, poorly differentiated

**Table 2:** Clinicopathological features of 40 cases for validation purpose

Sample no.	Group	Age	Gender	Virus infection status	Child-Pugh classification	Adjacent liver tissue	AFP (ng/ml)	TNM stage^a^	Tumor number	Tumor size (cm)	Differentiation^b^	Portal vein invasion	Intrahepatic metastasis	Duration of recurrence (year)
41	S	47	M	HBV	A	CH	242	3B	Multiple	11.50	Mod	Presence	Presence	0.28
42	S	37	F	HBV	A	CH	102,490	3A	Multiple	6.50	Por	Presence	Absence	1.30
43	S	65	M	None	A	CH	3,104,000	4A	Single	9.80	Por	Presence	Absence	0.34
44	S	55	M	HBV	A	CH	23.2	2	Single	6.70	Mod	Presence	Presence	0.43
45	S	57	M	HCV	A	CH	4718	2	Single	4.50	Por	Presence	Presence	0.43
46	S	52	M	HCV	A	LC	25	2	Single	5.80	Mod	Presence	Presence	1.05
47	S	62	M	HBV	A	CH	62	2	Single	7.00	Mod	Presence	Absence	0.41
48	S	79	M	HCV	A	CH	134	3A	Multiple	5.00	Por	Presence	Presence	0.70
49	S	67	M	HCV	A	CH	18.7	2	Multiple	4.00	Mod	Presence	Absence	0.61
50	S	66	M	HCV	A	CH	2970	2	Multiple	3.50	Por	Presence	Presence	0.58
51	L	53	M	None	B	LC	43.5	1	Single	3.50	Mod	Absence	Absence	2.98
52	L	71	M	None	A	Normal	2.7	1	Single	3.50	Well	Absence	Absence	3.45
53	L	58	M	None	A	Normal	7.5	1	Single	5.80	Mod	Absence	Absence	8.14
54	L	61	M	HBV	A	CH	6.4	1	Single	2.70	Well	Absence	Absence	5.37
55	L	78	M	HCV	A	CH	8.4	1	Single	2.70	Mod	Absence	Absence	5.42
56	L	60	M	HBV	A	CH	7.2	1	Single	2.60	Mod	Absence	Absence	2.71
57	L	64	F	HCV	A	LC	9.9	1	Single	3.60	Mod	Absence	Absence	2.23
58	L	64	M	HBV	A	CH	16.9	1	Single	3.90	Mod	Absence	Absence	4.61
59	L	72	M	HCV	B	CH	103.7	1	Single	7.40	Mod	Absence	Absence	3.72
60	L	74	M	None	A	Normal	4.2	1	Single	3.50	Mod	Absence	Absence	4.28
61	A	75	M	HCV	–	CH	–	–	–	–	–	–	–	–
62	A	65	F	HBV	–	LC	–	–	–	–	–	–	–	–
63	A	70	M	None	–	Normal	–	–	–	–	–	–	–	–
64	A	63	F	HBV	–	LC	–	–	–	–	–	–	–	–
65	A	85	M	HCV	–	CH	–	–	–	–	–	–	–	–
66	A	41	M	HBV	–	CH	–	–	–	–	–	–	–	–
67	A	58	M	HCV	–	CH	–	–	–	–	–	–	–	–
68	A	58	M	HBV	–	CH	–	–	–	–	–	–	–	–
69	A	73	M	None	–	CH	–	–	–	–	–	–	–	–
70	A	74	M	None	–	Normal	–	–	–	–	–	–	–	–
71	N	66	F	None	–	Normal	–	–	–	–	–	–	–	–
72	N	69	M	None	–	Normal	–	–	–	–	–	–	–	–
73	N	60	M	None	–	Normal	–	–	–	–	–	–	–	–
74	N	73	M	None	–	Normal	–	–	–	–	–	–	–	–
75	N	63	M	None	–	Normal	–	–	–	–	–	–	–	–
76	N	59	M	None	–	Normal	–	–	–	–	–	–	–	–
77	N	65	M	None	–	Normal	–	–	–	–	–	–	–	–
78	N	47	F	None	–	Normal	–	–	–	–	–	–	–	–
79	N	68	F	None	–	Normal	–	–	–	–	–	–	–	–
80	N	71	M	None	–	Normal	–	–	–	–	–	–	–	–

*S* duration of recurrence less than 2 years, *L* duration of recurrence more than 2 years, *A* adjacent liver tissue, *N* normal liver tissue, *HBV* hepatitis B virus, *HCV* hepatitis C virus, *CH* chronic hepatitis, *LC* liver cirrhosis

^a^TNM Classification of Malignant Tumours, 7th Edition, Sobin LH, Wittekind Ch (eds): International Union Against Cancer (UICC): ‘‘TNM classification of malignant tumors.’’ 7th ed. New York

^b^Well, well differentiated; Mod, moderately differentiated; Por, poorly differentiated

### Protein extraction

Proteins were extracted from surgically resected tissues as previously reported [19]. In brief, the frozen tissues were powdered in liquid nitrogen using metal beads (Multi-beads shocker; Yasui-kikai, Osaka, Japan). The tissues were then treated with urea lysis buffer (2 M thiourea, 6 M urea, 3% CHAPS, and 1% Triton X-100). After centrifugation, the supernatant was recovered as a soluble protein fraction and stored at −80 °C until use.

### Western blotting and image analysis

Protein expression levels were examined by western blotting. Five micrograms of protein were separated by sodium dodecyl sulfate polyacrylamide gel electrophoresis (SDS-PAGE) with various acrylamide concentrations according to the expected molecular mass of target proteins (ATTO, Tokyo, Japan). The separated proteins were transferred to a nitrocellulose membrane, which was reacted with primary antibodies. We selected 192 proteins as those associated with apoptosis, according to the pathway maps in MetaCore (GeneGo, St. Joseph, MI, USA) and Kyoto Encyclopedia of Genes and Genomes (KEGG; http://www.genome.jp/kegg). A list of 247 antibodies against the 192 proteins along with the providers’ codes and their dilutions is provided in Additional file 1: Table S1. The dilutions of antibodies were determined according to the manufacturer’s instructions. An antibody for actin (A5060; Sigma-Aldrich, St. Louis, MO, USA) was used at 1:250 dilution as a loading control. A horseradish peroxidase-conjugated antibody (GE Biosciences, Uppsala, Sweden) was used at 1:1000 dilution to detect the immuno-complex. The signal was visualized by enhanced chemiluminescence (ECL Plus; GE Biosciences) and an LAS-3000 system (GE Biosciences). The intensity of protein bands was measured using ImageQuant image analysis software (GE Biosciences). Membrane-to-membrane variations were normalized to the intensity of the actin band.

### Statistical analysis

Overall survival and disease-free survival curves were generated using the Kaplan–Meier method [20]. Statistical analyses were performed using SPSS software (SPSS Inc., IBM, Chicago, IL, USA).

## Results

To obtain the global expression profiles of apoptosis-associated proteins, we examined the surgically resected tissues by western blotting. We selected 192 proteins based on the contents of MetaCore and KEGG pathway maps, for which 247 antibodies were used. Fifty-one of these antibodies recognized different epitopes of the same protein (Additional file 1: Table S1).

**Fig. 1:**
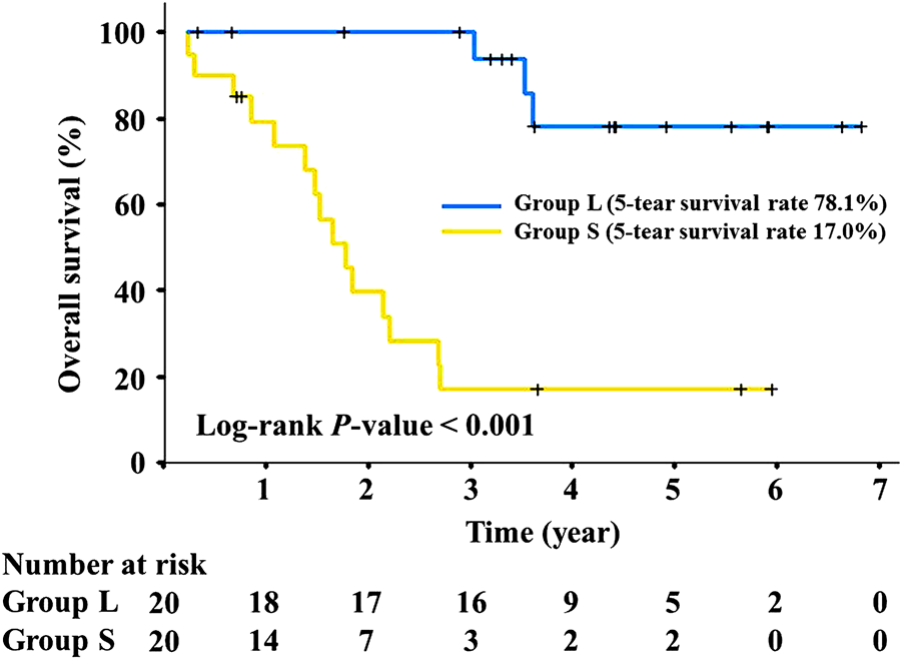
Survival curves of the 40 patients with HCC included in this study. Overall survival is shown for the two groups of patients: group S did and group L did not have recurrence within 2 years of surgery (n = 20 each). The two groups presented distinct prognoses

We examined the survival of patients with HCC and confirmed that patients with early recurrence presented shorter survival than those without recurrence (Fig. 1). These observations were in agreement with previous reports that the recurrence-free period is a critical prognostic factor for HCC [21].

**Fig. 2:**
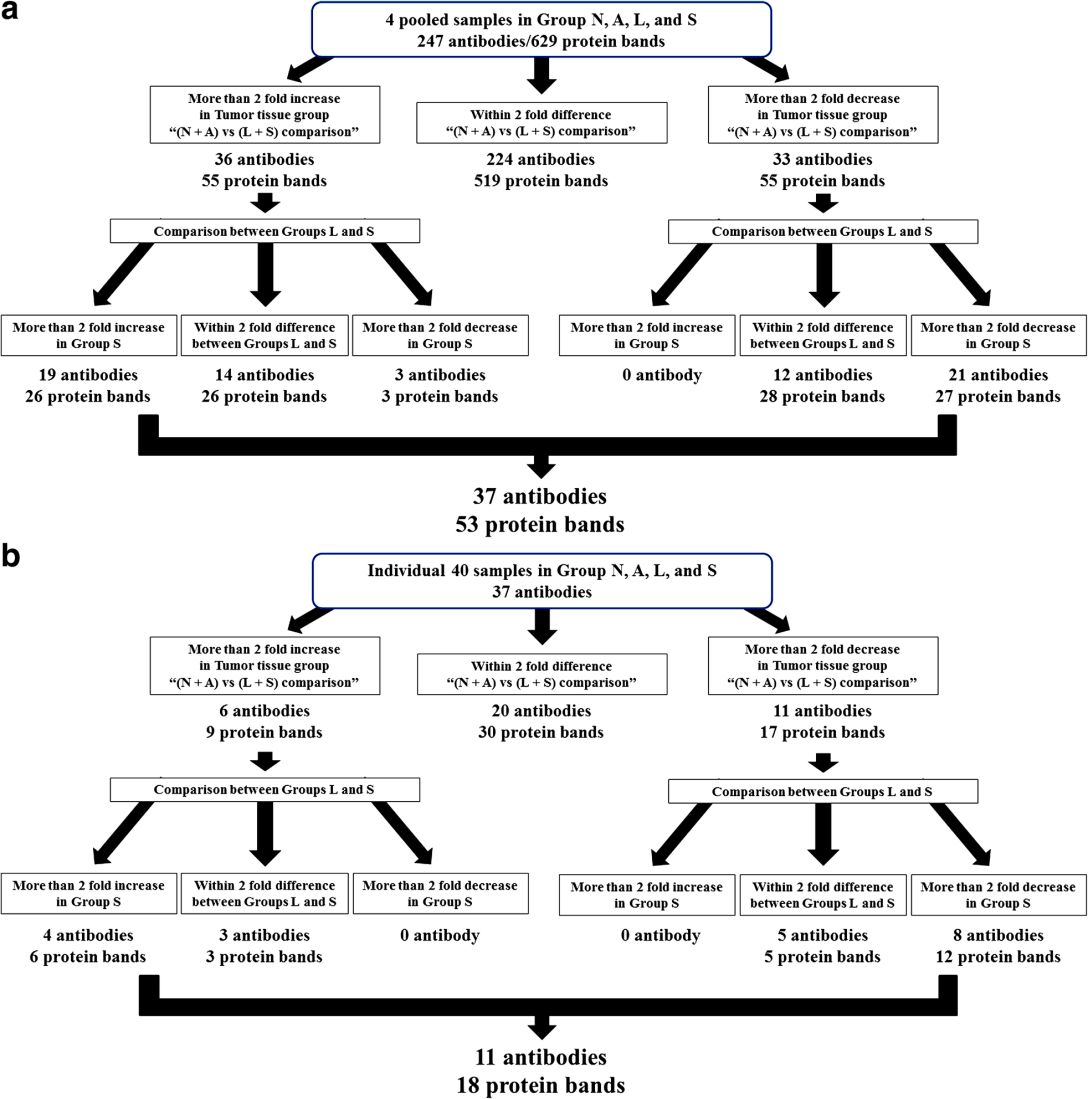
Flow chart of the experimental procedure and results of first and second screenings. A panel of 247 antibodies for 192 apoptosis-associated proteins was screened in surgically resected HCC and adjacent normal tissue samples. Single antibodies generated multiple protein bands by western blotting. Band intensity was compared between samples.** a** First screening using four sample pools such as group N (normal liver tissue),* A* (adjacent liver tissue),* L* (tumor tissue without recurrence), and* S* (tumor tissue with recurrence within 2 years of surgery).** b** Second screening for the selected 37 antibodies

 The screening process to identify apoptosis-related proteins is illustrated in Fig. 2. In the first screening (Fig. 2a), we generated four sample pools consisting of 10 samples from each groups, group N (normal liver tissue), A (adjacent liver tissue), L (tumor tissue without recurrence), and S (tumor tissue with recurrence within 2 years of surgery). The four group samples were subjected to western blotting (Table 1). The 247 antibodies generated 629 protein bands in the pooled samples. For a comparative analysis, we merged groups N and A as the non-tumor group and Groups L and S as the tumor group. We selected protein bands that had >twofold intensity differences between these two groups. Examples of western blotting data are presented in Additional file 2: Figure S1. Fifty-five protein bands corresponding to 36 antibodies showed >twofold increased intensity and 55 bands corresponding to 33 antibodies showed >twofold decreased intensity in the tumor group. We compared groups L and S with respect to these 69 antibodies in order to identify proteins with concordant alterations during HCC carcinogenesis and progression. Twenty-six bands (19 antibodies) and 27 bands (21 antibodies) showed a >twofold increase and decrease in intensity, respectively, in group S as compared to group L. Antibodies generated multiple protein bands. The protein bands generated by the same antibodies appeared to increase or decrease in intensity in group S. After accounting for the overlap between these two sets of protein bands and antibodies, 53 protein bands and 37 unique antibodies were selected.

**Fig. 3:**
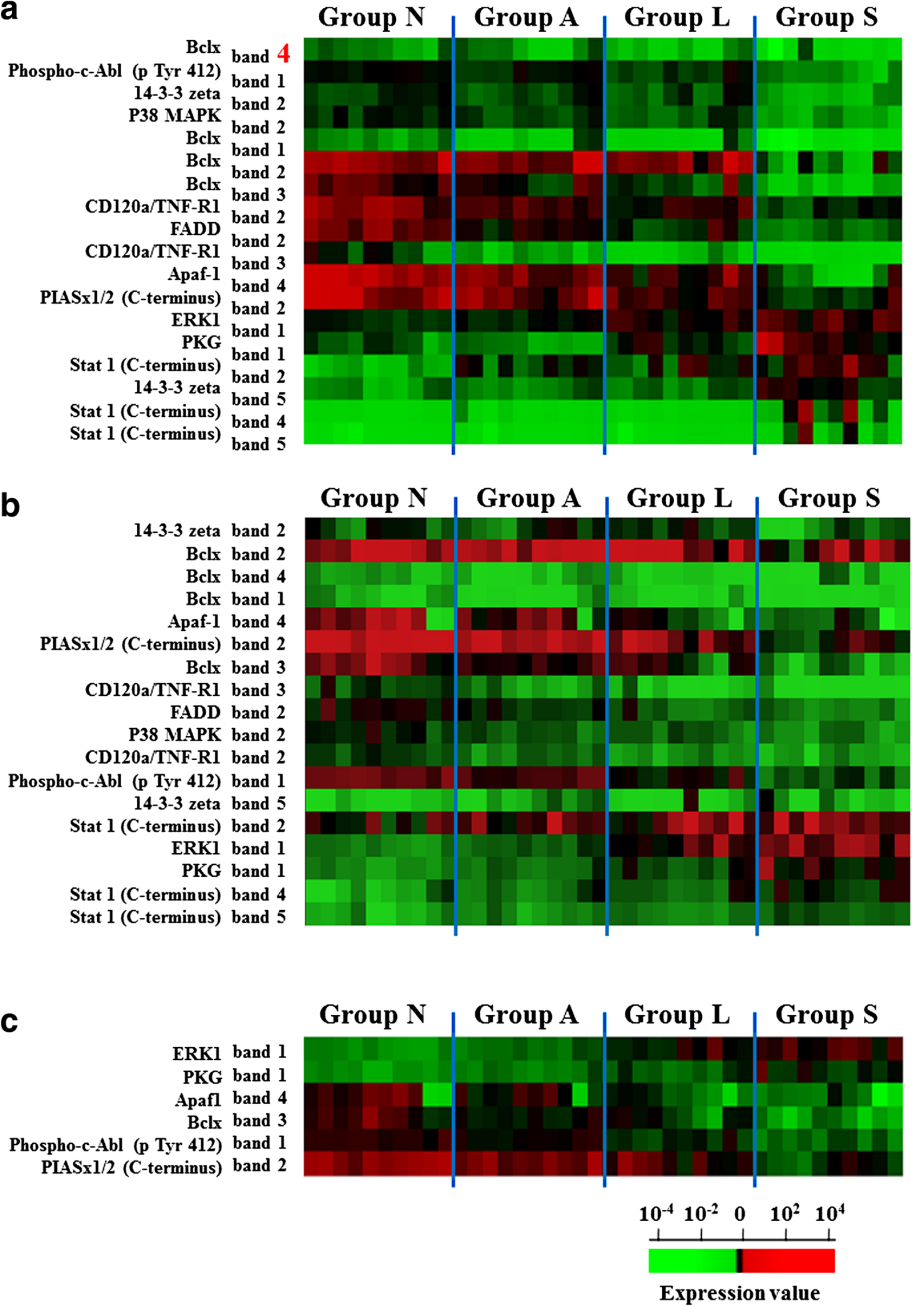
Heat map of antigens recognized by 11 selected antibodies. Band intensities were normalized among samples and blots. Measured intensities are displayed as a heat map. **a** In the first screening, the intensity of 18 protein bands for 11 antibodies was up- or downregulated in HCC carcinogenesis and progression. **b** In the next screening, we examined the selected 11 antibodies using the 40 samples from the newly enrolled patients. **c** In the independent validation study, the intensity of six bands for six antibodies differed among sample groups. The quantitative data for western blotting images are presented in Additional file 3: Table S2, Additional file 4: Table S3, Additional file 5: Table S4

For the second screening, we examined the individual samples used to generate the pooled samples and the selected 37 antibodies (Fig. 2b). The average intensity of nine protein bands (six antibodies) was increased, while that of 17 protein bands (11 antibodies) was decreased by >twofold in tumor tissues compared with non-tumor tissues (Table 3). In addition, the average intensity of protein bands by four antibodies was increased, while that by eight antibodies was decreased by >twofold in group S samples. After accounting for the overlap between these two sets of protein bands and antibodies, 18 protein bands and 11 unique antibodies were selected. The intensity levels of 18 protein bands detected by the 11 antibodies are illustrated in the heat maps (Fig. 3a).

**Table 3:** The 11 antibodies in western blotting screening

Protein name	Fold difference (Ratio of median)				Pathway
	Test set		Validation set		
	NA versus LS	L versus S	NA versus LS	L versus S	
Apaf-1 band 4	0.160	0.106	0.177	0.343	Caspase cascade
Bclx band 1	0.150	0.104	0.777	0.771	Mitochondrial pathway
Bclx band 2	0.283	0.050	0.622	0.377	Mitochondrial pathway
Bclx band 3	0.104	0.155	0.244	0.268	Mitochondrial pathway
Bclx band 4	0.220	0.214	0.746	0.913	Mitochondrial pathway
CD120a/TNF-R1 band 2	0.324	0.376	0.519	0.836	Extrinsic pathway
CD120a/TNF-R1 band 3	0.196	0.368	0.339	0.806	Extrinsic pathway
FADD band 2	0.176	0.395	0.408	0.721	Extrinsic pathway
p38MAPK band 2	0.402	0.404	0.576	0.722	Extrinsic pathway
phospho-c-Abl(pTyr412) band 1	0.362	0.413	0.295	0.475	Extrinsic pathway
PIASx1/2 (C-term) band 2	0.201	0.270	0.216	0.304	p53 banding pathway
14-3-3zeta band 2	0.499	0.328	0.774	0.453	p53 banding pathway
14-3-3zeta band 5	2.220	3.780	1.099	1.105	p53 banding pathway
ERK1 band 1	2.715	2.017	5.996	2.040	Extrinsic pathway
PKG band 1	3.874	2.548	2.635	2.027	Extrinsic pathway
Stat1(C-terminus) band 2	8.270	2.801	1.748	1.225	Caspase cascade
Stat1(C-terminus) band 4	2.058	6.237	2.119	1.696	Caspase cascade
Stat1(C-terminus) band 5	2.221	4.080	1.518	1.399	Caspase cascade

In the third screening, we examined the selected 11 antibodies using the 40 samples from the newly enrolled patients (Fig. 3b). The clinical and pathological data for the 40 patients are shown in Table 2. The average intensity of 4 protein bands (4 antibodies) was increased, while that of nine protein bands (six antibodies) was decreased by >twofold in tumor tissues compared with that in non-tumor tissues. In addition, the average intensity of two bands (two antibodies) was increased, while that of four bands (four antibodies) was decreased by >twofold in group S samples compared with those in group L. We demonstrated the intensity levels of six protein bands detected by the six antibodies are illustrated in the heat maps (Fig. 3c). The isoforms of six proteins included ERK1, PKG, Apaf1, BclX, phosphorylated c-abl, and PIASx1/2. The quantification of image intensity in Fig. 3 is summarized in Additional file 3: Table S2, Additional file 4: Table S3, Additional file 5: Table S4.

**Fig. 4:**
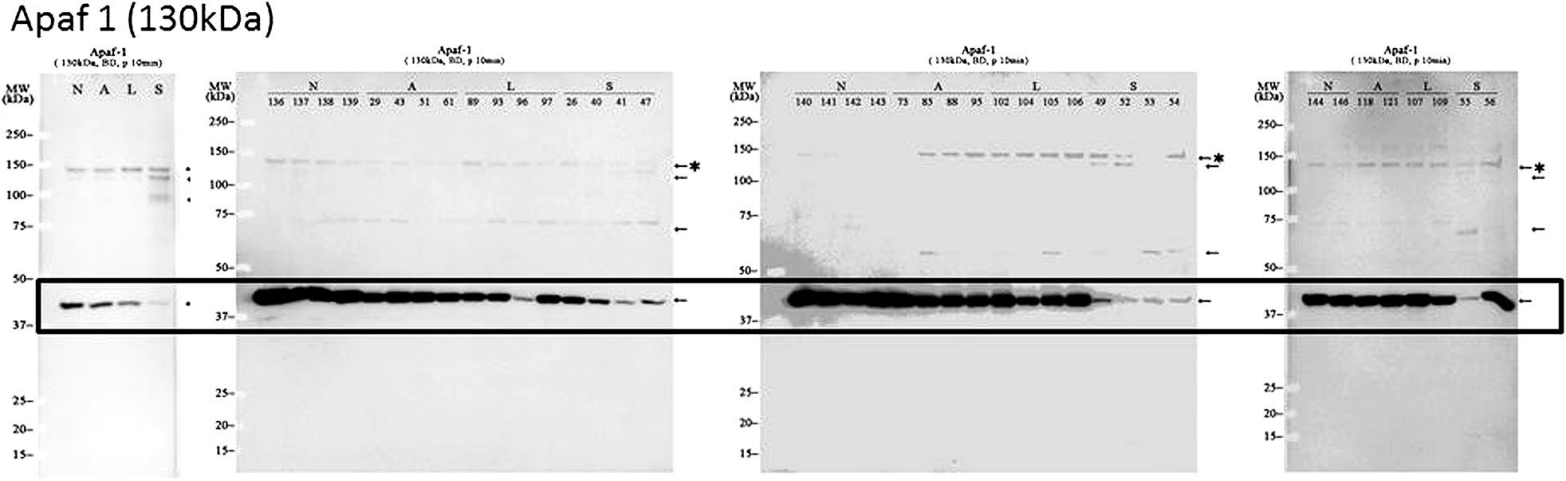
Western blot analysis of apaf-1 expression. Signals were observed in multiple locations in the blot and showed different intensities among sample groups. The theoretical molecular mass of apaf-1 is 130 kDa. The locations of the apaf-1 bands are indicated by *arrows*. The expected location of the apaf-1 band is indicated by an *asterisk*. The locations of apaf-1 bands associated with cancer progression and recurrence are *boxed*

We observed protein bands at unexpected locations on the gel, possibly resulting from posttranslational modifications such as phosphorylation and protein degradation. For example, apaf-1 showed an unexpected molecular mass of 40 kDa (Fig. 4). A 30-kDa isoform was previously reported in the biologically inactive apoptosome complex [22], indicating that the 40-kDa-apaf-1 protein band should be investigated in the context of HCC.

## Discussion

Early recurrence after surgical resection is a hallmark of poor prognosis in HCC and identifying the molecular components is critical for developing novel therapies. The association between apoptosis and HCC carcinogenesis and progression has previously been suggested, and proteins in the apoptosis pathway are considered as biomarkers as well as therapeutic targets [8–11]. In contrast to previous studies that examined these proteins individually, we adopted a comprehensive approach and examined a panel of 247 antibodies in order to clarify the molecular events underlying early recurrence.

In this study, we used samples stratified according to the different status of recurrence after curative surgery (Tables 1, 2). As the early recurrence is associated with malignant features of tumor tissues, the patients with early recurrence were at a more advanced stage (Tables 1, 2). Thus, the early recurrence biomarkers generated from this study are also those used for staging. Indeed, we detected significant correlations between early recurrence and poor prognosis (Fig. 1), and the proteins identified in this study may be associated with poor prognosis.

We used western blotting as a tool for examining protein expression. Western blotting allows the visualization of intact proteins using specific antibodies along with their isoforms with distinct molecular mass. Previous studies indicated that alternatively spliced isoforms of various proteins are associated with cancer phenotypes [23–27]. Western blotting has long been used in cancer research and may be particularly useful for studying aberrant protein isoforms, especially when used in combination with an antibody library.

Western blotting has drawbacks. Paramount among these is that the results may be affected by non-specific antibody reactions. In the present study, multiple bands corresponding to target proteins appeared at different locations than those predicted by their amino acid sequence. These were nonetheless included in the analysis since they do not necessarily reflect non-specific reactions; they may be novel cleavage fragments that have diagnostic utility. In a previous study, we reported an association between a novel variant of STAT3 and vascular invasion in HCC that was identified by SDS-PAGE combined with mass spectrometry [18], which can help validating western blotting data. The use of mass spectrometry and the different antibodies against the same proteins, but different epitopes, may be helpful to confirm the protein identity. The linear dynamic range of western blotting signal is also a subject of discussion. For wider dynamic range, the use of fluorescence-based detection may be worth considering.

We identify six proteins whose band intensities were associated with early recurrence in HCC (Fig. 3c). Those included ERK1 and Bclx, the aberrant regulations of which were associated with poor prognosis in HCC in previous studies [28–33]. Several ERK inhibitors are currently in clinical trials and the functional contributions of ERK families are worth investigating in HCC [34, 35]. Further validation studies of identified proteins are required for clinical applications.

In conclusion, we used a panel of antibodies against proteins in the apoptosis pathway to identify those associated with early HCC recurrence. Our results indicated that western blotting using specific antibodies can be a powerful tool to investigate the expression of selected protein groups and to capture the overall views of given molecular events in cancer. This approach can be applied to other protein groups in cancer proteomics. Western blot is the only method that separates the proteins according to their molecular mass and measures them individually with high sensitivity. Thus, the conventional clinical examination modalities such as IHC and ELISA may not be adequate as the modalities to apply the western blotting results to clinical applications. We may need to develop novel innovative clinical examination modalities, including a fully automated western blotting system.

## Additional files

**Additional file 1: Table S1.** List of antibodies examined in this study.

**Additional file 2: Figure S1.** Western blotting data of six proteins, which were associated with early recurrence in HCC.

**Additional file 3: Table S2.** Normalized intensity of protein bands in Individual 40 samples in Group N, A, L, and S.

**Additional file 4: Table S3.** Normalized intensity of protein bands in newly enrolled 40 samples in Group N, A, L, and S.

**Additional file 5: Table S4.** Normalized intensity of protein bands in newly enrolled 40 samples in Group N, A, L, and S.

## Abbreviations

abl: abelson murine leukemia viral oncogene homolog
Apaf-1: apoptotic protease-activating factor 1
Bcl2: B cell lymphoma 2
Bcl-xL: B cell lymphoma extra-large
ERK1: extracellular signal-regulated kinase 1
HCC: hepatocellular carcinoma
KEGG: Kyoto Encyclopedia of Genes and Genomes
MAPK: p38 mitogen-associated protein kinase
PIASx1/2: protein inhibitor of activated STAT1 and 2
PKG: protein kinase G
STAT1: signal transducer and activator of transcription 1
STAT3: signal transducer and activator of transcription 3
