# Supplementary figures and images for: Antibody-based proteomics to identify an apoptosis signature for early recurrence of hepatocellular carcinoma
Source: Clin Proteomics. 2016 Oct 24;13:28. doi: 10.1186/s12014-016-9130-0 (PMC5078925; doi:10.1186/s12014-016-9130-0)

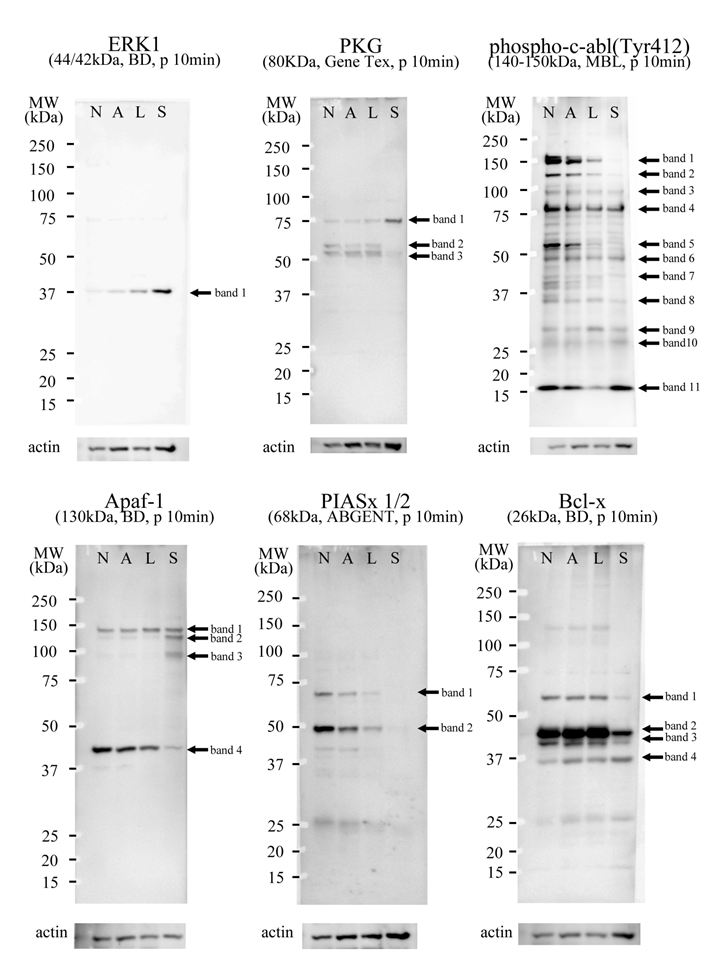

Supplement: Supplementary file 2 — Additional file 2: Figure S1. Western blotting data of six proteins, which were associated with early recurrence in HCC. [file 12014_2016_9130_MOESM2_ESM.tif]
